# Pellagra Forum

**Published:** 1916-08

**Authors:** 

**Affiliations:** Hot Springs, Ark.


### PELLAGRA FORUM.

DR. E. H. MARTIN, EDITOR, HOT SPRINGS, ARK.

Address all communications on this subject to Dr. Martin.

I have had some experience with the intravenous use of quinine dihydrochloride in two cases of pellagra recently.

I have frequently given quinine hydrobrom. after the method of Dr. Isadore Dyer, but have failed to get the good results which he obtains. Consequently I have not trusted a case of pellagra to quinine by the mouth for several years.

Recently a patient came in with pellagra and also gave a history of having had what seemed to him to be a malarial paroxysm each day for several days and at intervals previously for several weeks. Although the blood examination was negative for plasmodia I felt sure that he was suffering from malarial poisoning as well as pellagra. He was entirely too weak to begin any arsenical preparation or treatment which would produce a reaction, and owing to the amount of medicines which he had taken his stomach was useless for quinine. So he was given ten grains of dihydrochloride of quinine intravenously. Twenty-four hours later he had improved so much that this dose was repeated. He was given ten grains of quinine intravenously each day for seven days, at the end of which time he had gained nearly thirteen pounds, almost two pounds a day.

The skin lesions were mild in this case and nothing definite could be determined as to their progress. The diarrhea had checked and all fever had stopped. The very weak patient had become strong.

If I felt that all the above results were due to the effect of the quinine on *pellagra* I would have kept it up, but I could not but feel that it was due to the anti-malarial effect of the drug, and consequently the patient was then put on my routine treatment with salvarsan.

Another case which was making a very pleasing recovery under the use of salvarsan had developed the burning and pains in hands and feet which are so common in this disease. She had had four small doses of salvarsan and her diarrhea had been checked from twelve bowel movements a day to one or two. Her very severe skin lesions were scaling off rapidly. The bad taste and ropey viscid secretion from the mouth and throat had stopped, in fact, she was getting well rapidly but was suffering terribly from pains and burning in her hands and feet at night. This was usually accompanied by a slight rise in temperature. She was suffering greatly and had not slept for several nights, so I decided to give her quinine as an experiment.

The first intravenous dose (8 gr.) was given at her bedside at 10 p. m. and the next morning she said she had never had any one dose of medicine to do her as much good; that she had slept well all night without any pain or burning. The third dose was administered yesterday, and today she seems to be improving very rapidly.

This patient is from Louisiana and may have had some masked malaria but no plasmodia could be found, and it may be that, after all, quinine has some specific effect on pellagra.

I have failed utterly to find any such effect when quinine is given by the mouth, but will follow up its intravenous use in other cases and hope to have more experiences of my own and others to report next month. E. H. M.

July 31, 1916.

